# Elevated PTK6 expression is associated with tumor immune microenvironment remodeling and predicts poor prognosis in endometrial carcinoma

**DOI:** 10.3389/fmed.2026.1842564

**Published:** 2026-06-03

**Authors:** Zebiao Ma, Jiongyu Chen, Lihua Tang, Jing Wang, Shuaibing Yang, Haituo Wu, Li Zhou, Qingsong Qin, Xiaojing Wang

**Affiliations:** 1 Department of Gynecologic Oncology, Guangdong Engineering Research Center of AI-Powered Precision Cancer Diagnostics and Therapeutics (Proposed)/GuangDong Engineering Technology Research Center of AI-Powered Precision Cancer Diagnostics and Therapeutics, Cancer Hospital of Shantou University Medical College, Shantou, China; 2 Laboratory of Human Virology and Oncology, Shantou University Medical College, Shantou, China; 3 Central Laboratory, Cancer Hospital of Shantou University Medical College, Shantou, China; 4 Department of Obstetrics and Gynecology, Second Affiliated Hospital of Shantou University Medical College, Shantou, China

**Keywords:** immunotherapy, prognostic biomarker, protein tyrosine kinase 6, tumor immune microenvironment, uterine corpus endometrial carcinoma

## Abstract

**Background:**

Uterine corpus endometrial carcinoma (UCEC) incidence rises globally, with >50% of advanced/recurrent patients showing poor response to immune checkpoint inhibitors due to inadequate biomarkers. Protein tyrosine kinase 6 (PTK6), aberrantly expressed in malignancies and linked to tumor progression, lacks defined clinical significance in UCEC.

**Methods:**

Multi-omics analysis utilized UCEC RNA-seq (TCGA/GEO) and proteomics (CPTAC). PTK6 protein interactors from STRING were used for functional enrichment analysis. Tumor microenvironment (TME) was assessed via ESTIMATE (stromal/immune scores), TIMER3 (immune infiltration), and TCIA (immunophenoscore, IPS). Immunohistochemistry (IHC) validation (*n* = 200) explored the prognostic value.

**Results:**

PTK6 was upregulated in UCEC (*p* < 0.001) with promoter hypomethylation (median β: 0.32 vs. normal 0.52, *p* < 0.001), highest in microsatellite instability-high (MSI-H) subtype, and correlated with tumor mutational burden (TMB). High PTK6 expression associated with worse overall survival (OS) in both entire cohort (HR = 2.065, 95% CI 1.060–4.203, *p* = 0.033) and MSI-H subtype (HR = 8.562, 95% CI 2.226–32.930, *p* = 0.0018). Exploratory analysis suggested low PTK6-linked reduced progression-free survival in POLE subtype (HR = 10.48, 95% CI 1.063–103.4, *p* = 0.044; wide CI suggests caution). PTK6-related genes were mainly involved in ERBB signaling, PD-1/PD-L1 immune checkpoint in cancers, and focal adhesion. Upregulated PTK6 was associated with an altered TME (increased neutrophils, decreased CD8^+^ T cells, reduced IPS; all *p* < 0.05). IHC confirmed PTK6 overexpression as an independent poor prognostic factor for OS (HR = 5.050, 95% CI 2.136–11.943, *p* < 0.001), associating with aggressive clinicopathological features (all *p* < 0.05).

**Conclusion:**

PTK6 represents a context-dependent prognostic biomarker linked to tumor immune microenvironment remodeling in UCEC, suggesting its potential utility for immunotherapeutic optimization.

## 1 Introduction

Uterine corpus endometrial carcinoma (UCEC), also termed endometrial cancer or endometrial adenocarcinoma, ranked as the sixth most common malignancy among women globally in 2022 (1). In the United States, UCEC mortality rates have risen more rapidly than those of any other female genital tract cancer (2), while its incidence has increased steadily at approximately 1% per year since the mid-2000s (3). According to the World Health Organization (WHO) Classification of Tumors of the Female Genital Tract (4th edition), it classifies carcinomas under a dual framework: Type I (estrogen-dependent endometrioid adenocarcinoma) and Type II (non-estrogen-dependent, including serous carcinoma and other aggressive histologies) (4). The Cancer Genome Atlas (TCGA) delineated four major molecular subgroups: DNA polymerase epsilon-ultramutated (POLE), microsatellite instability-high (MSI-H), copy number-low (CN-L), and copy number-high (CN-H) (5). Notably, MSI-H is a characteristic molecular phenotype resulting from deficiency in the DNA mismatch repair (dMMR) pathway (6).

Immune checkpoint inhibitors (ICIs) targeting MSI-H/dMMR tumors, have demonstrated significant efficacy in MSI-H/dMMR endometrial carcinomas. However, more than 50% of patients with advanced or recurrent UCEC derive no benefit from ICI treatment, as evidenced by objective response rates (ORR) of only 43.5–46% in clinical trials (7–10). Although the U. S. Food and Drug Administration (FDA) has approved predictive biomarkers for ICI efficacy, including programmed death-ligand 1 (PD-L1) expression, tumor mutational burden (TMB), and MSI-H/dMMR status (11), the lack of validated biomarkers hinders the precise selection of patients for immunotherapy (6).

Protein tyrosine kinase 6 (PTK6, also known as BRK and SIK) is a non-receptor tyrosine kinase with autophosphorylation capacity. Structurally similar to SRC family kinases, it contains a short unique amino terminus followed by SH3, SH2, and catalytic domain, but lacks the N-terminal myristoylation essential for membrane anchoring (12). PTK6 is predominantly expressed in epithelial tissues and overexpressed in multiple malignancies, including breast (13, 14), prostate (15, 16), colorectal (17, 18), head and neck (19), thyroid (20), pancreatic (21), hepatocellular (22, 23), cervical (24), and bladder cancer (25–27), where elevated PTK6 expression correlates with poor prognosis and promotes cancer cell migration, invasion and metastasis.

The prognostic significance of PTK6 in UCEC remains unexplored. In this study we investigated its clinical value. We first established its expression patterns and prognostic value through integrated analysis of multi-omics databases, including TCGA, Gene Expression Omnibus (GEO), and Clinical Proteomic Tumor Analysis Consortium (CPTAC). Subsequently, we identified correlations between PTK6 overexpression and an altered tumor microenvironment, suggesting its potential value as a candidate marker for immunotherapeutic stratification. Finally, we validated its prognostic value using immunohistochemistry (IHC).

## 2 Materials and methods

### 2.1 Dataset acquisition

Clinical characteristics and corresponding mRNA expression profiles of 529 UCEC samples were retrieved from the TCGA PanCancer Atlas dataset (containing MSI MANTIS scores, molecular subtypes, and TMB), downloaded via cBioPortal (RRID: SCR_014555; https://www.cbioportal.org/; accessed 11 October 2025). Matched transcriptome-derived transcripts per million (TPM) expression matrices were obtained from the UCSC Xena Browser (RRID: SCR_018938; https://xenabrowser.net/; accessed 11 October 2025). The GSE183185 dataset (GPL20795 platform, containing 30 paired tumor and adjacent normal endometrial tissues) was acquired from GEO (RRID: SCR_005012; https://www.ncbi.nlm.nih.gov/geo/; accessed 22 September 2025).

### 2.2 PTK6 expression in normal and cancer tissues across pan-cancer

The differential mRNA expression profile of PTK6 across 33 cancer types (including UCEC) between tumor and matched normal tissues was calculated by TIMER3, an online platform for systematic profiling of tumor immune infiltration (28) (https://compbio.cn/timer3/; accessed 12 October 2025). Promoter methylation levels and proteomic expression (CPTAC-derived data) of PTK6 in UCEC were obtained via UALCAN, an interactive web resource for multi-omics cancer data mining (29) (RRID: SCR_015827; https://ualcan.path.uab.edu/; accessed 20 October 2025). Scatter plots visualizing the correlations between MSI-MANTIS scores and PTK6 expression were generated using GraphPad Prism (GraphPad Software, Inc., Version 8.0.2; RRID: SCR_002798). The UCEC cohort was stratified by FDA-endorsed TMB thresholds (TMB-H: >10 mutations/Mb; TMB-L: ≤10 mutations/Mb) (11) to examine differential PTK6 expression across subgroups.

### 2.3 Prognostic value of PTK6 in the UCEC-TCGA cohort

Based on optimal expression cutoffs determined by X-tile (Version 3.6.1; RRID: SCR_005602), UCEC samples were dichotomized into high- and low-expression cohorts. Kaplan–Meier survival analyses were performed for the entire cohort and the four TCGA molecular subtypes to assess differences in overall survival (OS) and progression-free survival (PFS) between the two groups. TCGA-defined stratification (5) was applied to evaluate the prognostic value of PTK6 across distinct clinical subgroups.

### 2.4 Protein–protein interaction (PPI) network and enrichment analysis of PTK6

Protein co-expression relationships of PTK6 in *Homo sapiens* were extracted from STRING database (v12.0; RRID: SCR_005223; https://string-db.org/; accessed 12 November 2025) with a high-confidence interaction threshold set at a combined score ≥ 0.7. The PPI network of 27 node proteins centered on PTK6 was visualized via Cytoscape (v3.10.4; RRID: SCR_003032). Functional enrichment analysis of PPI-associated genes was performed using the Gene Ontology (GO) and Kyoto Encyclopedia of Genes and Genomes (KEGG) pathway databases, with statistical significance defined as a false discovery rate (FDR) < 0.05.

### 2.5 Correlation between PTK6 expression and tumor immune microenvironment features in UCEC

Stromal and immune scores were downloaded from the ESTIMATE algorithm (RRID: SCR_026090; https://bioinformatics.mdanderson.org/estimate; accessed 27 October 2025), a website designed to estimate stromal and immune cells in malignant tumor tissues using expression data.

Tumor immune dysfunction and exclusion (TIDE) scores, as defined by the TIDE algorithm (30), were calculated using the TIMER3 (accessed 22 October 2025) based on TCGA RNA-Seq data. Similarly, TIMER3 was used to assess correlations between PTK6 expression and infiltration of multiple immune cell types, evaluated through multiple algorithms: gene set enrichment-based approaches (xCell, MCP-counter); deconvolution-based computational approaches (CIBERSORT, EPIC, quanTIseq); and a deep profiling approach for T-cell subpopulations (ImmuCellAI).

Immunophenoscores (IPS) and immune gene sets for UCEC were downloaded from The Cancer Immunome Atlas (TCIA) database (31) (RRID: SCR_014508; https://tcia.at/; accessed 22 October 2025). A high IPS serves as a positive predictor for immunotherapy efficacy. The cutoff of 2.36 TPM was determined by the median value of PTK6 expression in the cohort for IPS correlation analysis. Other genes co-expressed with PTK6 for the consensus co-expression analysis were sourced from the GEPIA2 (32) (RRID: SCR_026154; http://gepia2.cancer-pku.cn/; accessed 28 October 2025) and UALCAN datasets (accessed 28 October 2025).

### 2.6 Tissue sample and patient information

The study was conducted in accordance with the Declaration of Helsinki, and approved by the Ethics Committee of the Cancer Hospital of Shantou University Medical College (Approval No. 2021038). We collected 200 de-identified formalin-fixed paraffin-embedded primary UCEC tissue samples from patients who underwent surgical treatment at this institution between 2002 and 2012. Serial 4-μm sections were prepared for IHC staining. Tumor histology and staging followed WHO classification and International Federation of Gynecology and Obstetrics (FIGO) 2009 staging criteria. Postoperative follow-up was conducted through standardized clinical and laboratory examinations on a standardized schedule: quarterly for the first 2 years, semiannually for the subsequent 3 years, and then annually for up to 5 years, until patient death or last visit. OS was calculated from the date of surgery to death or the last follow-up, while PFS was defined as the interval from initial surgery to disease progression or the last confirmed follow-up visit. Comprehensive clinical data extracted from medical records are presented in Table 1.

**Table 1:** Relationship between PTK6 IHC scores and clinicopathological features of UCEC patients.

Features	Total *n* = 200	PTK6 IHC^a^		*χ* ^2^	*p-*value
		Low	High		
Age (years)				0.300	0.584
≤ 50	67 (33.5%)	40 (59.7%)	27 (40.3%)		
> 50	133 (66.5%)	74 (55.6%)	59 (44.4%)		
Menopause				0.649	0.420
Pre	85 (42.5%)	52 (60.5%)	34 (39.5%)		
Post	115 (57.5%)	63 (54.8%)	52 (45.2%)		
Stage (FIGO 2009)				5.915	0.015*
I/II	137 (68.5%)	86 (62.8%)	51 (37.2%)		
III/IV	63 (31.5%)	28 (44.4%)	35 (55.6%)		
Grade				3.689	0.158
1	43 (21.5%)	30 (69.8%)	13 (30.2%)		
2	128 (64.0%)	69 (53.9%)	59 (46.1%)		
3	29 (14.5%)	15 (51.7%)	14 (48.3%)		
Lymph node				9.941	0.002*
Positive	38 (19.0%)	13 (34.2%)	25 (65.8%)		
Negative	162 (81.0%)	101 (62.3%)	61 (37.7%)		
Myometrial invasion				2.843	0.092
≤ 1/2	147 (73.5%)	89 (60.5%)	58 (39.5%)		
> 1/2	53 (26.5%)	25 (47.2%)	28 (52.8%)		
Cervical invasion				6.867	0.009*
No	167 (83.5%)	102 (61.1%)	65 (38.9%)		
Yes	33 (16.5%)	12 (36.4%)	21 (63.6%)		
Adnexal involvement				0.159	0.690
No	177 (88.5%)	100 (56.5%)	77 (43.5%)		
Yes	23 (11.5%)	14 (60.9%)	9 (39.1%)		
Ascites				-	0.734^**b**^
Positive	8 (4.00%)	4 (50.0%)	4 (50.0%)		
Negative	177 (88.5%)	99 (55.9%)	78 (44.1%)		
NA	15 (7.50%)	11 (73.3%)	4 (26.7%)		
Metastasis				15.931	< 0.001*
No	133 (66.5%)	89 (66.9%)	44 (33.1%)		
Yes	67 (33.5%)	25 (37.3%)	42 (62.7%)		
Adjuvant therapy					
Yes	84 (42.0%)	-	-		
Chemotherapy	35	-	-		
Radiotherapy	6	-	-		
Combination ^c^	43	-	-		
No	116 (58.0%)	-	-		

^a^ Low: score ≤ 6; High: score > 6; ^b^ Fisher’s exact Test; ^c^ chemotherapy + radiotherapy.

*P < 0.05. IHC, immunohistochemistry; UCEC, uterine corpus endometrial carcinoma; FIGO, international federation of gynecology and obstetrics.

### 2.7 IHC and semi-quantitative analysis

IHC analysis was performed on paraffin sections. After baking at 60 °C for 4 h, sections were deparaffinized with xylene and rehydrated through graded ethanol series (100, 95, 80, 70%). Following three rinses with phosphate-buffered saline (PBS), antigen retrieval was performed using citrate-hydrochloric acid buffer (pH 6.0) with microwave heating for 15 min. Endogenous peroxidase activity was quenched with 0.3% H2O2 for 15 min at room temperature, followed by PBS rinses and blocking with 5% normal sheep serum for 30 min. Sections were incubated overnight at 4 °C with a rabbit recombinant monoclonal PTK6 antibody (Abcam Cat# ab233392; RRID: AB_3741044), and then with HRP-conjugated secondary antibody (Cell Signaling Technology Cat# 8114, RRID: AB_10544930) for 30 min at room temperature. After PBS washes, color development was performed with 3,3′-diaminobenzidine (DAB) chromogen, followed by hematoxylin counterstaining. PTK6 expression was semi-quantitatively assessed by two blinded pathologists using a composite score comprising of staining extent and staining intensity. Staining extent was graded as: 0 (<5% tumor cells), 1 (5–25%), 2 (26–50%), 3 (51–75%), 4 (>75%). Staining intensity was graded as: 0 (absent), 1 (faint yellow), 2 (moderate yellow-brown), and 3 (strong brown). The final IHC score was calculated by multiplying the staining extent (0–4) and intensity (0–3) subscores, generating composite scores from 0 to 12. Inter-observer agreement was good between the two pathologists, with a Cohen’s kappa coefficient of 0.778 (95% Confidence Interval [CI] 0.715–0.847; *p* < 0.001). Samples were stratified by the median final IHC score into low-expression (≤ 6) and high-expression (> 6) groups for subsequent analyses. Robustness was interrogated via secondary verification using the third-quartile threshold (Q3 score = 8), confirming consistency of survival associations.

### 2.8 Statistical analysis and visualization

Cutoff values of TCGA-UCEC cohort were defined by X-tile (Version 3.6.1) (33). Statistical analyses were performed using SPSS 25.0 (IBM Corp.).

The prognostic significance of PTK6 in UCEC was evaluated via Kaplan–Meier analysis evaluated with log-rank tests. Univariable Cox regression models were used to identify predictors associated with OS, and covariates with *p* ≤ 0.25 were included in multivariable Cox regression analysis. Due to substantial heterogeneity in adjuvant treatment regimens, this factor was not incorporated as a covariate. Before Cox regression analysis, multicollinearity among all covariates was assessed using the variance inflation factor (VIF). A VIF value >10 was defined as severe multicollinearity, which can lead to misleading statistical results (34). The results showed that the VIF values of FIGO stage and its constituent factors (lymph node metastasis, depth of myometrial invasion, cervical invasion) were all less than 5, indicating no significant multicollinearity among variables. Therefore, all variables were included in the univariate and multivariate Cox proportional hazards regression models to identify independent prognostic factors for OS.

Associations between PTK6 expression and clinical parameters were analyzed using χ^2^ tests, while intergroup differences in continuous variables were assessed using Student’s t-tests. Fisher’s exact test was necessitated by low event rates (*n* < 5) in the ascites-positive subset. Pairwise multiple comparisons were performed using one-way ANOVA with Dunnett’s *post hoc* testing. Correlations between variables were analyzed using Spearman’s coefficients. Statistical significance was defined as *p* < 0.05. Data visualization was performed using the online platform (https://www.bioinformatics.com.cn; last accessed 31 December 2025).

## 3 Results

### 3.1 PTK6 is aberrantly expressed in pan-cancer and upregulated in UCEC with promoter hypomethylation

**Figure 1:**
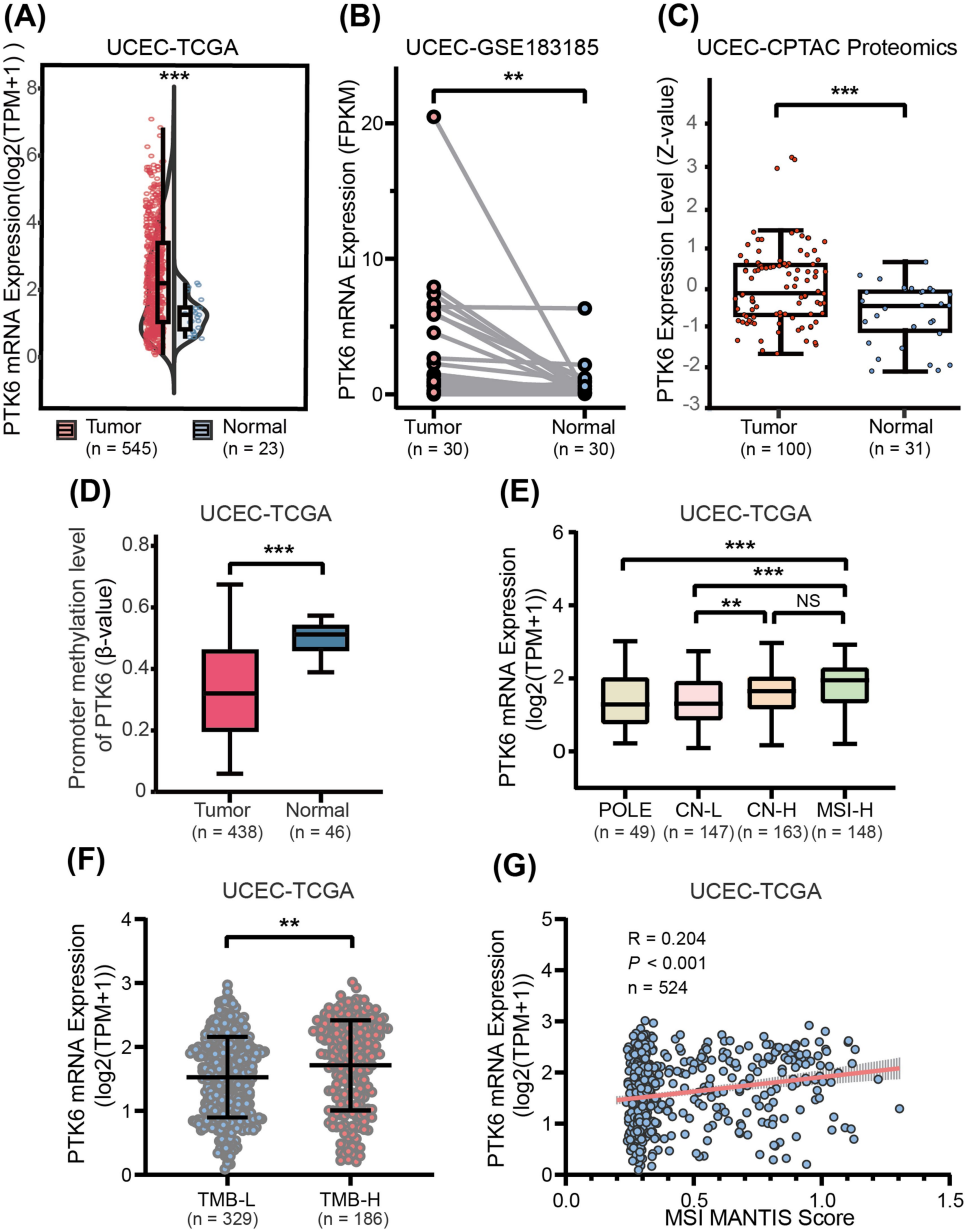
PTK6 is upregulated in UCEC with hypomethylation. **(A,B)** Differential *PTK6* mRNA expression in UCEC vs. paired normal tissues in **(A)** TCGA (TIMER3) and **(B)** GSE183185. **(C)** PTK6 protein overexpression in UCEC vs. normal tissues (UALCAN CPTAC proteomics; median *Z*-values: −0.057 vs. −0.706). **(D)** Promoter hypomethylation of *PTK6* in UCEC vs. normal tissues (UALCAN; median β-value: 0.32 vs. 0.52). **(E)** *PTK6* mRNA expression among four UCEC molecular subtypes. **(F)** *PTK6* expression in high-TMB and low-TMB groups. **(G)** Correlation between *PTK6* mRNA expression and MSI MANTIS score. *p < 0.05; **p < 0.01; ***p < 0.001.

A pan-cancer transcriptome screen revealed PTK6 mRNA dysregulation across malignancies: significantly upregulated in ten cancers (including UCEC) but downregulated in colon adenocarcinoma (COAD), kidney chromophobe carcinoma (KICH) and head and neck squamous cell carcinoma (HNSC) compared with paired normal tissues (Supplementary Figure S1). Focusing on endometrial carcinoma, comprehensive multi-omics analyses confirmed significant PTK6 upregulation. PTK6 mRNA expression was markedly higher in UCEC tissues than in normal controls in the TCGA cohort (*n* = 545 vs. 23, *p* < 0.001, Figure 1A) and the GSE183185 paired cohort (*n* = 30, *p* = 0.0075, Figure 1B). Congruently, mass spectrometry-based proteomics analysis from CPTAC confirmed PTK6 protein overexpression in UCEC (median Z-values = −0.057 vs. −0.706, *p* < 0.001, Figure 1C).

Mechanistically, UCEC tumors (*n* = 438) exhibited significant PTK6 promoter hypomethylation compared with normal tissues (*n* = 46), as evidenced by a reduced median *β*-value (0.32 vs. 0.52; *p* < 0.001, Figure 1D), with hypomethylation defined by established thresholds (β < 0.3) (35, 36). Furthermore, PTK6 expression varied significantly among UCEC molecular subtypes (highest in the MSI-H subtype, Figure 1E) and was weakly positively correlated with MSI score (R = 0.204, *p* < 0.001, Figure 1G). Expanding to genomic instability metrics, PTK6 expression was higher in the high-TMB group (TMB-H, ≥ 10mutations/Mb, *n* = 186) than in the low-TMB group (TMB-L, < 10mutations/Mb, *n* = 329; *p* = 0.002; Figure 1F).

### 3.2 PTK6 overexpression predicts adverse survival in key UCEC molecular subtypes

**Figure 2:**
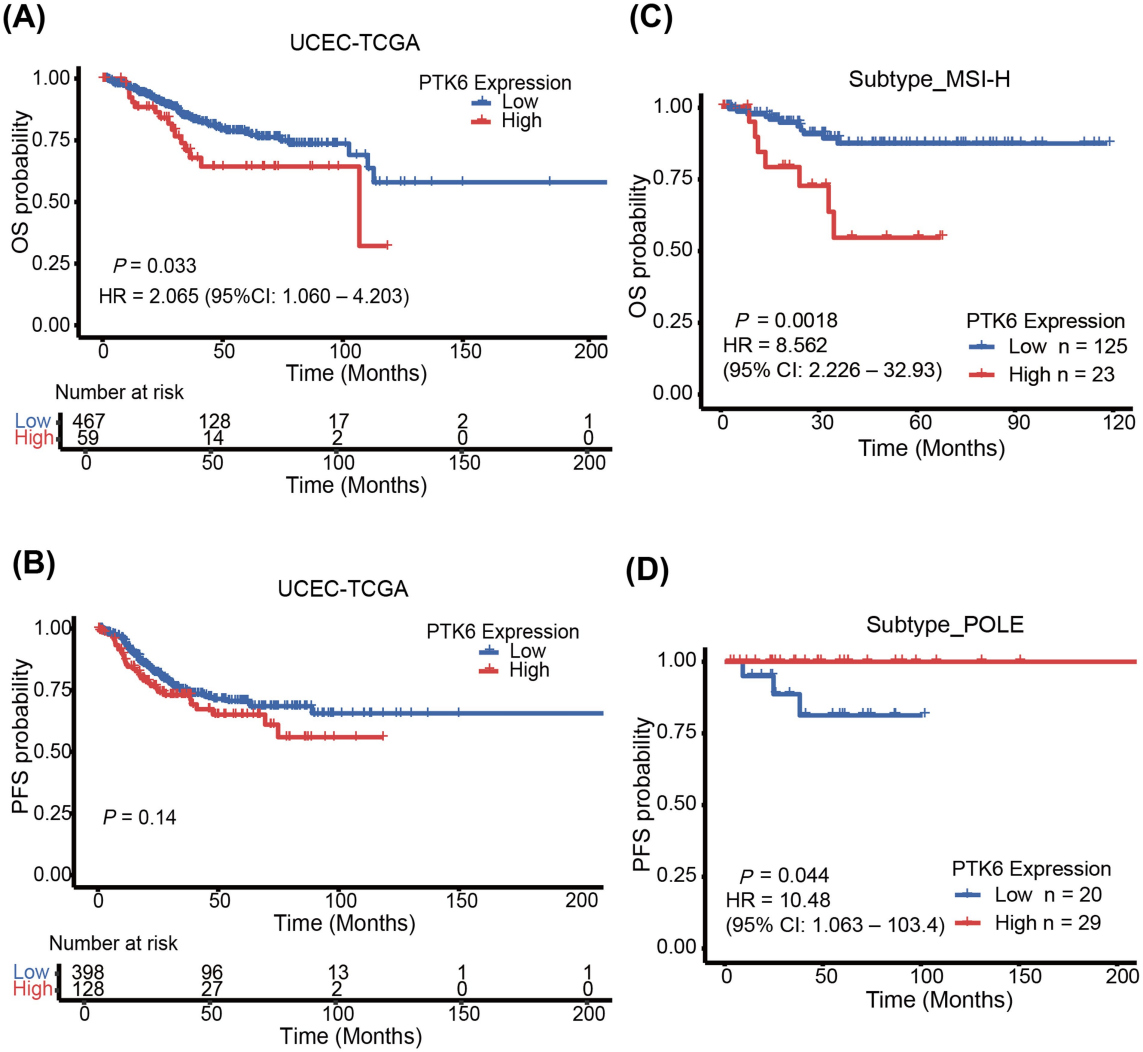
High PTK6 expression predicts poor survival in specific UCEC subtypes. Kaplan–Meier survival analyses based on PTK6 expression in the TCGA-UCEC cohort. **(A)** Overall survival (OS) and **(B)** Progression-free survival (PFS) in the total cohort. **(C)** OS in the MSI-H subtype. **(D)** PFS in the POLE subtype. Tables show patients at risk over time. HR: Hazard ratio; CI: Confidence interval.

Clinically, Kaplan–Meier analysis demonstrated that high PTK6 mRNA predicted worse OS in all UCEC patients [Hazard Ratio (HR) = 2.065, 95% CI: 1.060–4.203, *p* = 0.033, Figure 2A], although no significant difference in PFS was observed between the high- and low-expression groups (*n* = 59 vs. 398; *p* = 0.14, Figure 2B). Another cutoff validation strategy in the UALCAN database consistently established significant associations between elevated PTK6 expression and adverse OS outcomes (*p* = 0.014, Supplementary Figure S5A). Notably, stratification by molecular subtypes revealed striking associations: high PTK6 expression in MSI-H tumors conferred an 8.6-fold increased risk of death (*p* = 0.0018, Figure 2C); conversely, low PTK6 expression in POLE tumors (*n* = 49) predicted shorter PFS (HR = 10.48, 95% CI: 1.063–103.4, *p* = 0.044, Figure 2D). The prognostic effect of PTK6 in this subtype is exploratory owing to small sample size and extremely wide CI, and thus requires cautious interpretation. In contrast, CN-H and CN-L subtypes showed no significant differences both in OS and PFS (Supplementary Figure S2).

### 3.3 PTK6 interaction network implicates cancer-associated pathways

**Figure 3:**
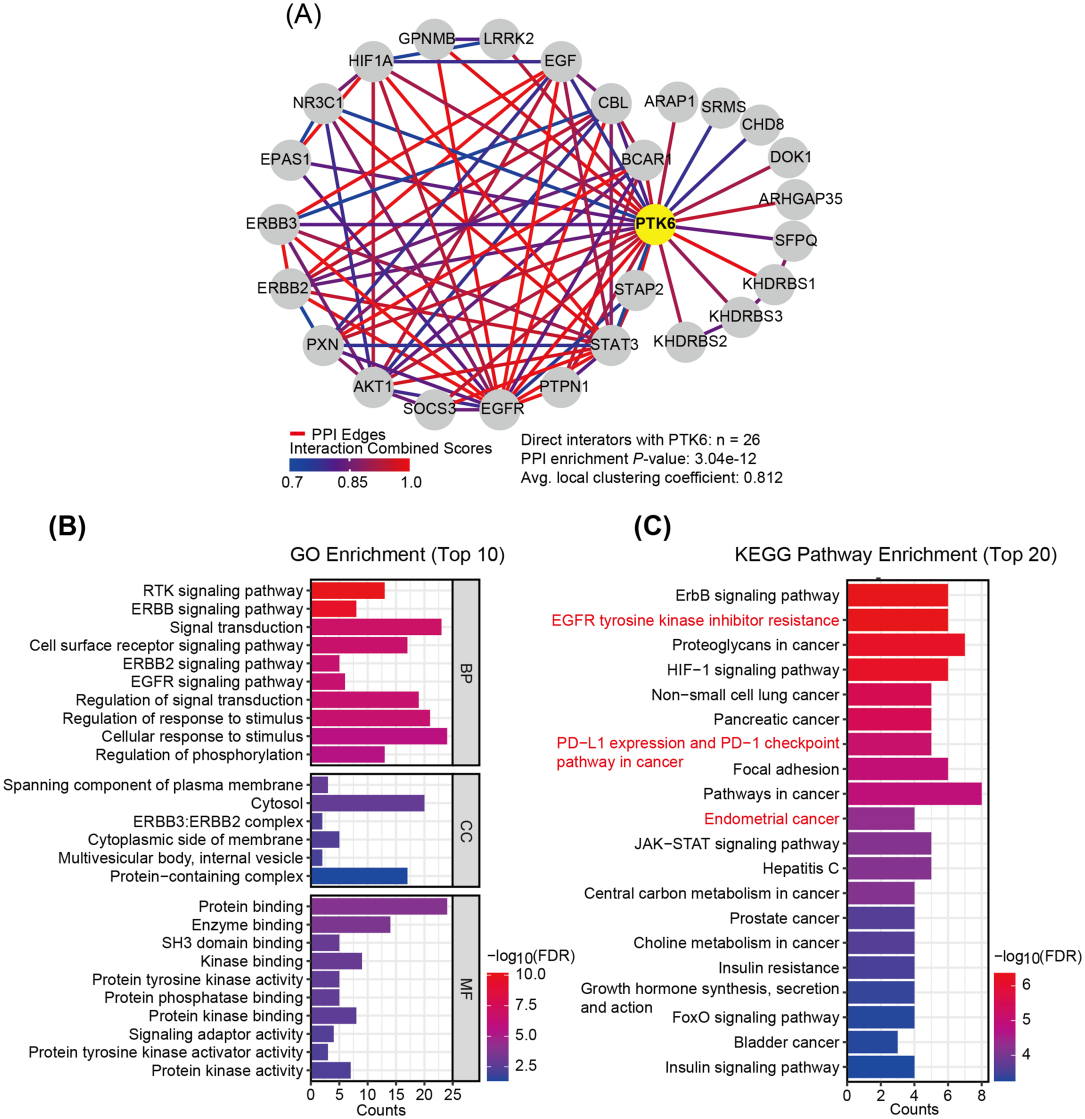
PTK6 interaction network and functional enrichment. **(A)** Protein–protein interaction (PPI) network of PTK6 and its 26 direct interactors (STRING v12.0, confidence ≥ 0.7). **(B)** Top 10 Gene Ontology (GO) enrichment terms, including biological process (BP), cellular component (CC), molecular function (MF). **(C)** Top 20 KEGG pathway enrichment results. Focused cancer-associated pathways are highlighted. Bar lengths represent gene count; color indicates −log10(FDR). RTK, transmembrane receptor protein tyrosine kinase; EGFR, epidermal growth factor receptor.

STRING analysis identified 26 high-confidence (combined score ≥ 0.7) PTK6 interactors, forming a densely connected network (PPI enrichment *p* = 3.04e-12, Figure 3A). Functional enrichment linked PTK6 to critical oncogenic processes, including the ERBB signaling pathway (GO:0038127, FDR < 0.001, Figure 3B), EGFR tyrosine kinase inhibitor resistance (KEGG:01521), focal adhesion (KEGG:04510), PD-L1 expression and PD-1 checkpoint pathway in cancer (KEGG:05235), proteoglycans in cancer (KEGG:05205) and endometrial cancer (KEGG:05213) (Figure 3C). These results suggest that PTK6 may act as a key signaling hub regulating tumor invasion and tumor microenvironment crosstalk in UCEC.

### 3.4 PTK6 overexpression is associated with an altered tumor microenvironment in UCEC

**Figure 4:**
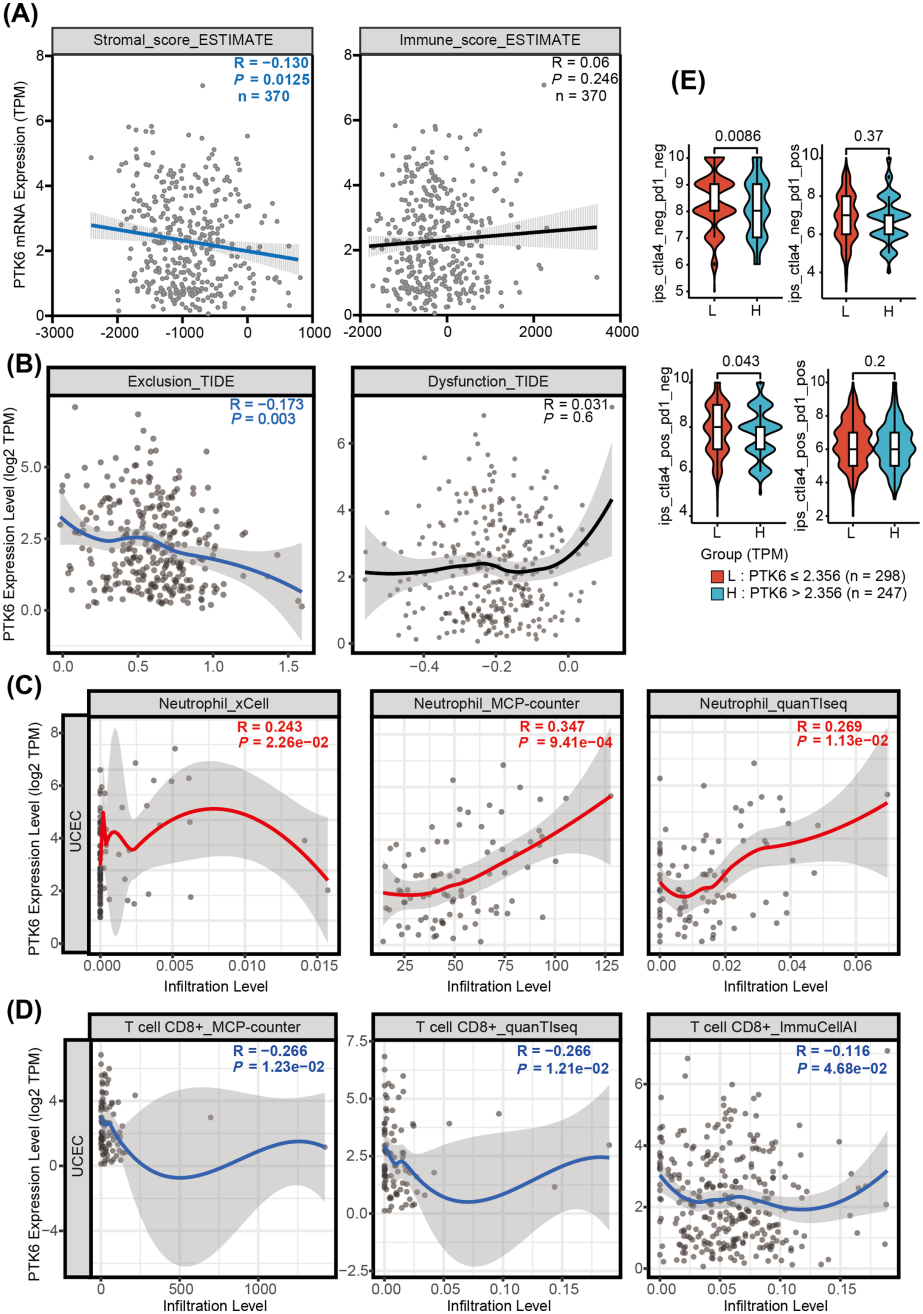
PTK6 expression correlates with tumor microenvironment remodeling in UCEC. **(A)** Left: Negative correlation with stromal score (*R* = −0.130, *p* = 0.0125). Right: No correlation with immune score (*p* > 0.05). **(B)** Left: Negative correlation with TIDE exclusion score (*R* = −0.173, *p* = 0.003). Right: No correlation with TIDE dysfunction score (*p* > 0.05). **(C,D)** PTK6 positively correlates with **(C)** neutrophil infiltration and negatively with **(D)** CD8^+^ T cell infiltration across multiple algorithms. **(E)** Immunophenoscore (IPS) in low vs. high PTK6 expression groups: reduced in the CTLA4^−^PD-1^−^ (*p* = 0.0086) and CTLA4^+^PD-1^−^ (*p* = 0.043) groups under high PTK6 expression.

To further explore the relationship between PTK6 expression and the tumor immune microenvironment (TIME) in endometrial cancer, we analyzed the correlation between PTK6 mRNA expression and stromal/immune scores (ESTIMATE algorithm), as well as TIDE exclusion/dysfunction scores (TIDE algorithm) in the TCGA-UCEC cohort. As shown in Figure 4A, PTK6 expression was significantly negatively correlated with the stromal score (Spearman’s rank correlation coefficient R = −0.130, *p* = 0.0125, *n* = 370), indicating that higher PTK6 expression was associated with a lower stromal component in the TIME. In contrast, no significant correlation was observed between PTK6 expression and the immune score (R = 0.06, *p* = 0.246, *n* = 370), suggesting that PTK6 may not directly affect the overall level of immune cell infiltration in endometrial cancer.

Subsequently, we evaluated the impact of PTK6 on T-cell function using the TIDE algorithm. Notably, PTK6 expression was significantly negatively correlated with the TIDE exclusion score (R = −0.173, *p* = 0.003, Figure 4B left), implying that elevated PTK6 expression might reduce the likelihood of T-cell exclusion from the tumor microenvironment. However, no significant association was found between PTK6 expression and the TIDE dysfunction score (R = 0.031, *p* = 0.6, Figure 4B right), indicating that PTK6 may not directly influence the functional state of tumor-infiltrating T cells.

We further demonstrated the relationship between PTK6 expression and immune cell infiltration level in UCEC using multiple immune infiltration analysis methods (Supplementary Figure S3). Consistent positive correlations between neutrophil infiltration level and PTK6 expression were observed across three independent analytical methods (xCell/MCP-counter/quanTIseq: R = 0.243/0.347/0.269, all *p* < 0.05, Figure 4C), while CD8 + T-cell infiltration levels showed negative correlations with its expression (MCP-counter/quanTIseq/ImmuCellAI: R = −0.266/−0.266/−0.116, all *p* < 0.05, Figure 4D). Critically, high PTK6 expression was associated with reduced potential for immune checkpoint inhibitor response, as evidenced by lower IPS in CTLA4^−^PD-1^−^ (*p* = 0.0086) and CTLA4^+^PD-1^−^ (*p* = 0.043) subgroups (Figure 4E). Six immune-related genes (C2CA4A, CDA, TNFSF9, FUT4, TNFRSF14 and IL4R) tightly correlated with PTK6 were identified through consensus co-expression analysis of UCEC immune gene sets from the TCIA database, integrated with UALCAN and GEPIA2 datasets, and subsequent validation via TIMER3 (all *p* < 0.05, Supplementary Figure S4).

### 3.5 Elevated PTK6 expression serves as a novel independent prognostic biomarker in UCEC

To evaluate the clinical value of PTK6, we retrospectively analyzed 200 UCEC patients stratified by PTK6 IHC expression (low: score ≤ 6; high: score > 6). High PTK6 expression was significantly correlated with aggressive clinicopathological features, including advanced FIGO 2009 stage (III/IV vs. I/II: 55.6% vs. 37.2%, *p* = 0.015), positive lymph node metastasis (65.8% vs. 37.7%, *p* = 0.002), cervical invasion (63.6% vs. 38.9%, *p* = 0.009), and distant metastasis (62.7% vs. 33.1%, *p* < 0.001). No significant associations were observed between PTK6 expression and age, menopausal status, tumor grade, depth of myometrial invasion, adnexal involvement, or ascites (*p* > 0.05) (Table 1).

**Figure 5:**
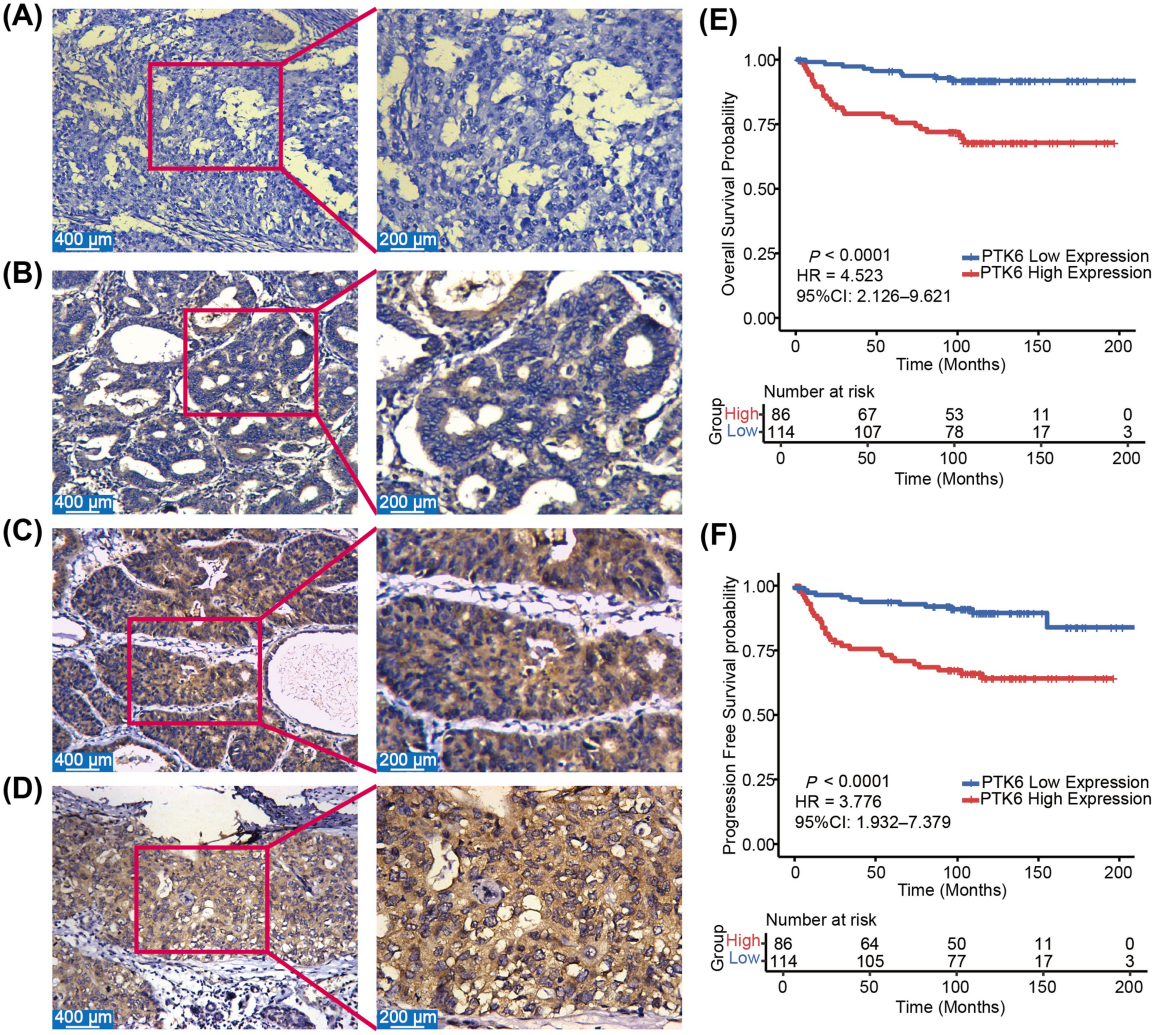
PTK6 protein expression validates prognostic significance in UCEC. **(A–D)** Representative IHC staining of PTK6 in UCEC tissues: **(A)** Low, **(B)** intermediate, **(C)** high expression, and **(D)** positive control (in breast carcinoma tissue). Scale bars: left = 400 μm (overview), right = 200 μm (inset). **(E,F)** Kaplan–Meier curves showing that high PTK6 expression (IHC score > 6) correlates with shorter **(E)** OS and **(F)** PFS.

Univariate Cox regression analysis identified high PTK6 expression as a predictor of poor OS (HR = 4.523, 95% CI 2.126–9.621, *p* < 0.001), along with 10 other risk factors (*p* < 0.05) (Table 2). Multivariate Cox regression analysis confirmed that high PTK6 protein expression (HR = 5.050, 95% CI 2.136–11.943, *p* < 0.001), age (≥ 50 years, HR = 4.822, *p* = 0.023), lymph node metastasis (HR = 3.075, *p* = 0.0497), deep myometrial invasion (> 1/2, HR = 2.308, *p* = 0.038), and ascites (HR = 4.164, *p* = 0.048) were independent predictors of poor OS. In contrast, FIGO stage, cervical invasion, and adnexal involvement lost statistical significance in the multivariate model (Table 2). IHC validation further demonstrated that patients with high PTK6 expression (> median) had significantly worse OS (HR = 4.523, *p* < 0.0001, Figure 5E) and PFS (HR = 3.776, *p* < 0.0001, Figure 5F) compared with those with low PTK6 expression, consolidating its clinical prognostic relevance. Importantly, this OS association persisted when analyzed with the more stringent Q3 cutoff (Q3 = 8; HR = 3.529, *p* < 0.0001; Supplementary Figure S5B), confirming the robustness of the prognostic association.

**Table 2:** Univariate and multivariate Cox regression analysis of overall survival in UCEC patients.

Variables	Univariate analysis			Multivariate analysis		
	HR	95% CI	*p*-value	HR	95% CI	*p*-value
Age (years)(< 50 vs. ≥ 50)	2.546	1.060–6.118	0.037*	4.822	1.285–18.086	0.020*
Menopause (pre vs. post)	2.235	1.051–4.753	0.037*	0.386	0.117–1.274	0.118
Stage (FIGO 2009) (I/II/III/IV)	3.062	2.139–4.383	< 0.001*	1.465	0.754–2.847	0.260
Grade (1/2/3)	1.727	0.992–3.007	0.053	1.607	0.757–3.412	0.217
Lymph node (positive vs. negative)	9.122	4.656–17.871	< 0.001*	3.075	1.001–9.439	0.0497*
Myometrial invasion (≤ 1/2 vs. > 1/2)	5.966	3.020–11.785	< 0.001*	2.308	1.045–5.097	0.038*
Cervical invasion (no vs. yes)	4.490	2.313–8.717	< 0.001*	2.263	0.917–5.584	0.076
Adnexal involvement (no vs. yes)	2.522	1.149–5.534	0.021*	0.902	0.273–2.984	0.866
Ascites (positive vs. negative)	8.656	1.966–38.104	0.004*	4.164	1.013–17.115	0.048*
Metastasis (no vs. yes)	6.171	2.974–12.805	< 0.001*	0.835	0.278–2.505	0.749
PTK6 expression (IHC ≤ 6 vs. IHC > 6)	4.523	2.126–9.621	< 0.001*	5.050	2.136–11.943	< 0.001*

*p < 0.05. UCEC, uterine corpus endometrial carcinoma; HR, hazard ratio; CI, confidence interval; FIGO, international federation of gynecology and obstetrics.

## 4 Discussion

Previous studies have confirmed that PTK6 overexpression predicts poor outcomes in breast, prostate, liver, and colorectal cancers (14, 17, 22, 37, 38). Our work extends these findings to UCEC, where multi-omics data show significant PTK6 upregulation at both the mRNA and protein levels (Figure 1). PTK6 expression varied by molecular subtype (highest in the MSI-H subtype) and was positively correlated with TMB. Notably, high PTK6 expression predicted worse survival in UCEC, particularly in the MSI-H subtype, where it was associated with an 8.6-fold increased risk of death (Figure 2). These results were validated in 200 tumor samples using our IHC scoring system (Figure 5). We also found reduced DNA methylation in the PTK6 promoter in UCEC, suggesting epigenetic regulation of PTK6 expression—a possible mechanism that requires further experimental validation.

Remarkably, PTK6 exerted opposing effects across UCEC molecular subtypes: high expression increased mortality risk in MSI-H tumors, whereas exploratory analysis in POLE cases suggested that low expression predicted shorter PFS. This context-dependent dichotomy aligns with prior reports: PTK6 inhibits growth in normal gut/skin cells but drives cancer progression in breast, liver and prostate tumors (15, 23, 37, 39). Intriguingly, studies have revealed that PTK6 has nuclear-cytoplasmic shuttling capability: cytoplasmic PTK6 exerts oncogenic effects in prostate and liver cancers, while nuclear localization is associated with tumor-suppressive activity (16, 23). Paradoxically, tumor-suppressive functions persist in specific cancers like esophageal (40, 41) and laryngeal squamous cell carcinoma (42).

Mechanistically, our results suggest that PTK6 acts as a signaling nexus in cancer-associated pathways, supported by high-confidence PPI networks (Figure 3) enriched in ERBB signaling, EGFR tyrosine kinase inhibitor resistance, PD-1/PD-L1 immune checkpoint regulation, and focal adhesion. These links are well-established in other malignancies: in colorectal cancer, PTK6 activates JAK2/STAT3 to maintain cancer stem cells and chemotherapy resistance (18); in prostate cancer, it triggers epithelial-mesenchymal transition (EMT) to promote tumor metastasis (15).

Importantly in UCEC, high PTK6 expression was associated with altered tumor microenvironment remodeling (Figure 4), characterized by reduced CD8 + T-cell infiltration, increased neutrophil recruitment, and lower indicators of ICI response represented by reduced IPS. Paradoxically, upregulated PTK6 was inversely correlated with the TIDE exclusion score, implying that the stromal microenvironment shaped by elevated PTK6 may be more permissive to T-cell entry. This observation underscores the complexity of the tumor immune microenvironment in endometrial carcinoma. To address this apparent discrepancy, we noted that PTK6 overexpression was positively associated with neutrophil infiltration, which was consistent with previous findings in bladder cancer, where PTK6 recruits neutrophils through CXCL1/8 signals to facilitate metastasis (27). Tumor-associated neutrophils (TANs) are heterogeneous and comprise the N1 subtype with anti-tumor activity and the N2 subtype with potent immunosuppressive functions (43). N2-polarized TANs are well-established suppressors of CD8 + T cells in the tumor microenvironment. They markedly impair CD8 + T-cell function, proliferation, and persistence, and may even induce T-cell exclusion, through multiple mechanisms including arginase-mediated arginine depletion (44), reactive oxygen species production, secretion of immunosuppressive factors including TGF-β and IL-10, and recruitment of regulatory T cells (45). Together, these results suggest that PTK6 may shape the tumor microenvironment by regulating stromal components and excluding T-cell infiltration, driving tumors toward a “cold tumor” phenotype and thus conferring resistance to immunotherapy (46).

Clinically, high PTK6 protein was associated with aggressive clinicopathological features, including advanced FIGO stage, lymph node/distant metastasis, and cervical invasion. Two independent cutoff validation strategies consistently confirmed that PTK6 overexpression was a significant predictor of inferior overall survival in the IHC cohort. Furthermore, it remained an independent prognostic factor for poor overall survival in multivariate analysis (HR = 5.050). Although FIGO stage was a prognostic factor in the univariate analysis, it lost statistical significance in the multivariate analysis (VIF < 5, excluding multicollinearity). This is likely due to the absorption of its prognostic information by more precise pathological components (lymph node metastasis, depth of myometrial invasion) and the adjustment for other strong prognostic factors in the multivariate model. PTK6 thus provides additional prognostic value beyond standard clinical staging.

However, our study has several limitations. First, it is a retrospective study, which is inherently subject to selection bias. Second, the lack of functional validation *in vitro* or *in vivo* leaves the underlying mechanisms by which PTK6 promotes tumor metastasis and interacts with immune cells in UCEC unresolved. Third, the prognostic effect of PTK6 in POLE subtype is exploratory owing to small sample size and extremely wide CI, and thus requires cautious interpretation. Fourth, molecular subtype data were not available in the IHC cohort, limiting subtype-specific validation in tissue samples. Fifth, the application of data-driven X-tile cutoffs may affect the generalizability of our findings.

Future research may enable the clinical translation of our findings: PTK6 IHC staining could complement existing biomarkers (MSI-H/dMMR, TMB) to optimize the selection of UCEC patients for immunotherapy. Furthermore, pharmacological inhibition of PTK6 might represent a potential strategy to remodel the tumor microenvironment and enhance the efficacy of ICIs in UCEC. This hypothesis requires validation in preclinical models and prospective clinical trials involving patients with endometrial cancer, particularly those resistant to ICIs.

## 5 Conclusion

In summary, our study establishes that PTK6 is upregulated in UCEC and acts as a context-dependent independent prognostic biomarker. PTK6 overexpression is associated with tumor microenvironment remodeling, characterized by altered immune cell infiltration. These findings support the potential utility of PTK6 for stratifying patients and optimizing immunotherapeutic strategies in UCEC, which warrants further prospective validation in ICI-treated cohorts.

## Data Availability

The original contributions presented in the study are included in the article/Supplementary material, further inquiries can be directed to the corresponding author.
