# Supplementary figures and images for: Elevated PTK6 expression is associated with tumor immune microenvironment remodeling and predicts poor prognosis in endometrial carcinoma
Source: Front Med (Lausanne). 2026 Jun 3;13:1842564. doi: 10.3389/fmed.2026.1842564 (PMC13272333; doi:10.3389/fmed.2026.1842564)

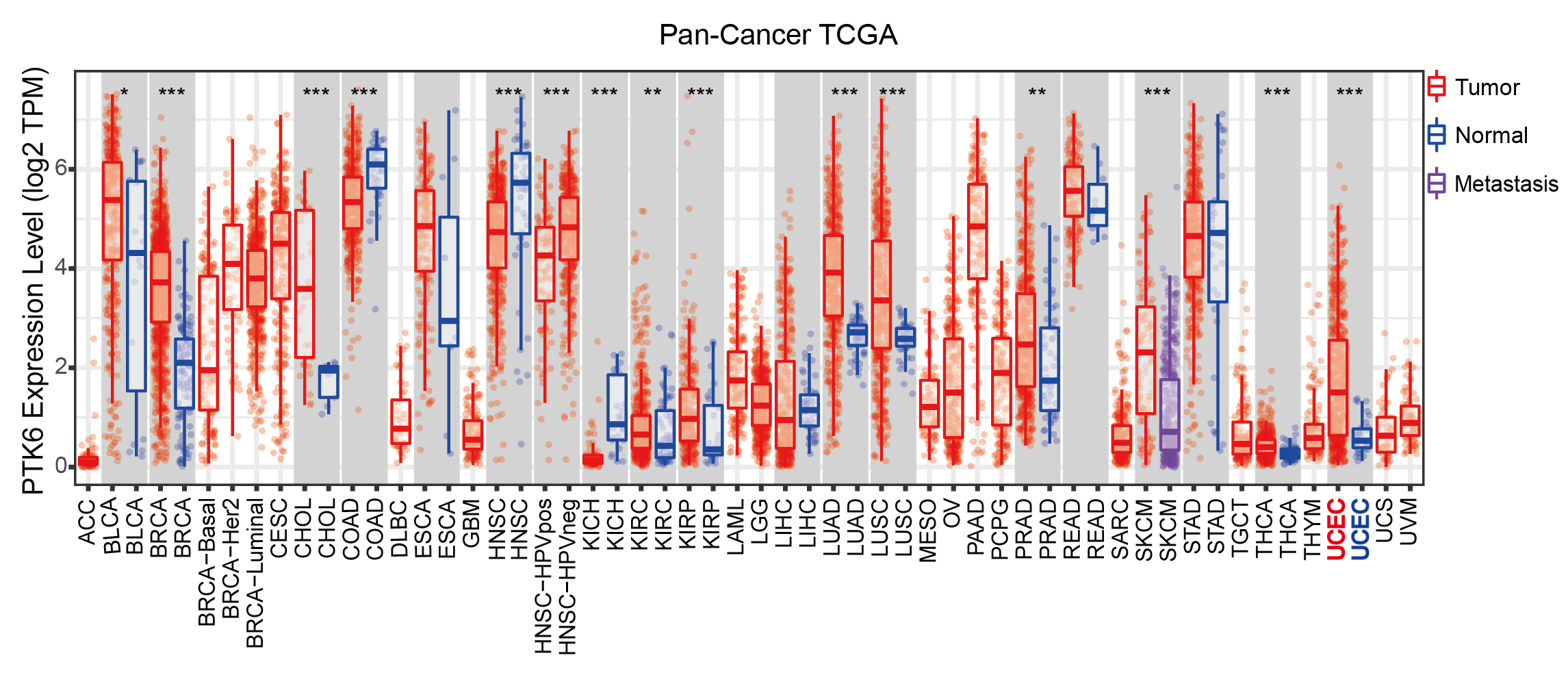

Supplement: Supplementary file 1 [file image_1.tif]

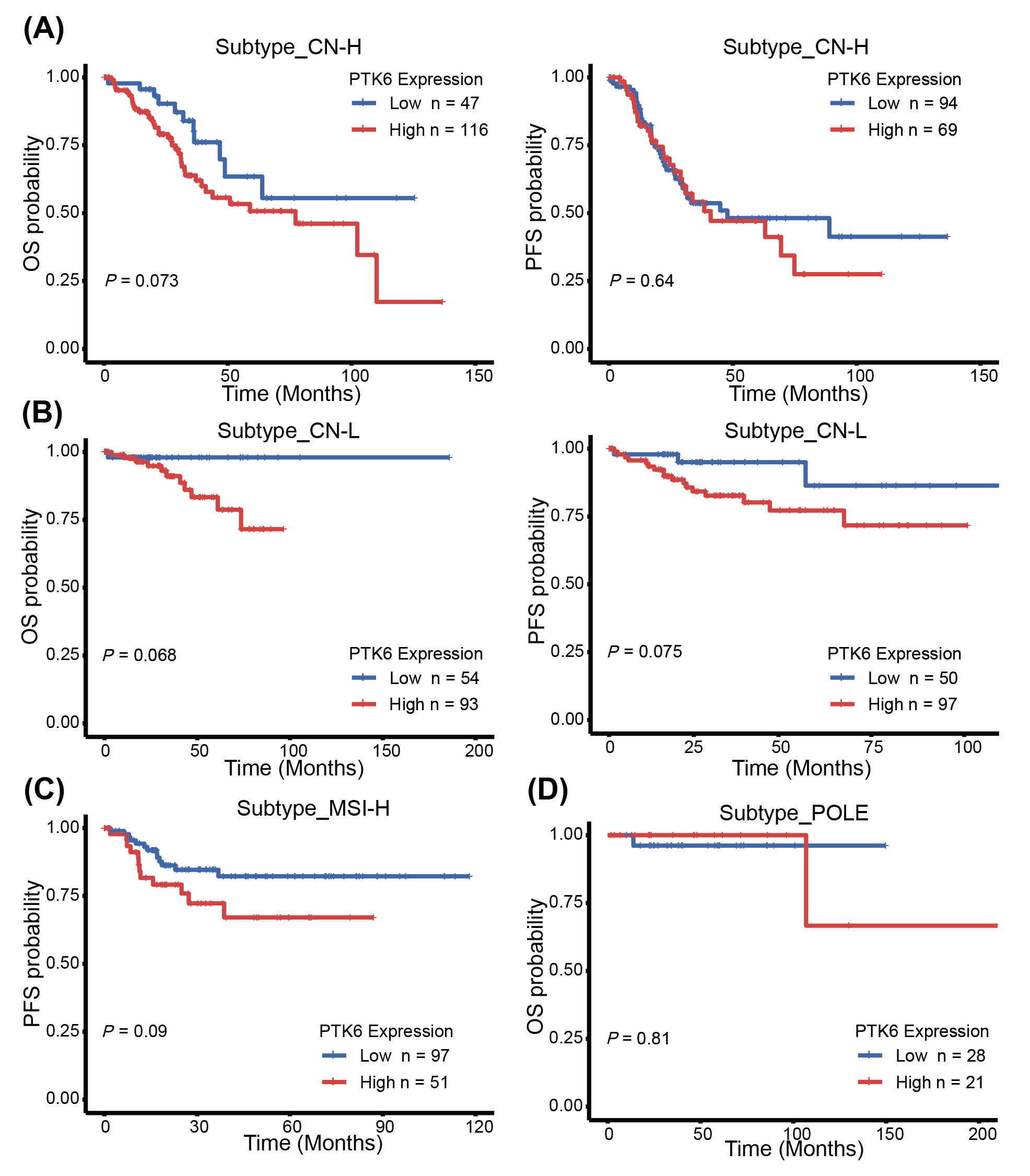

Supplement: Supplementary file 2 [file image_2.tif]

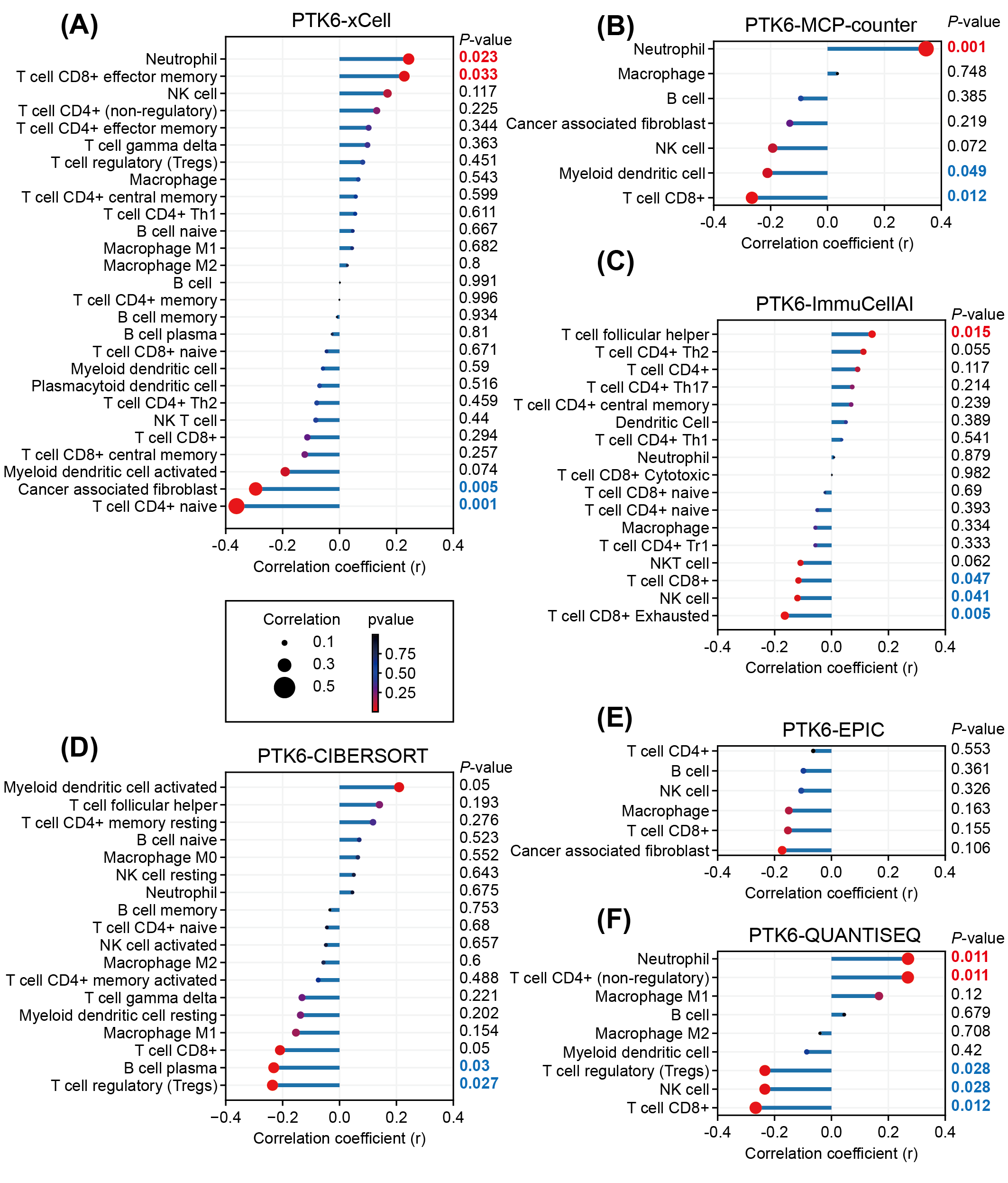

Supplement: Supplementary file 3 [file image_3.tif]

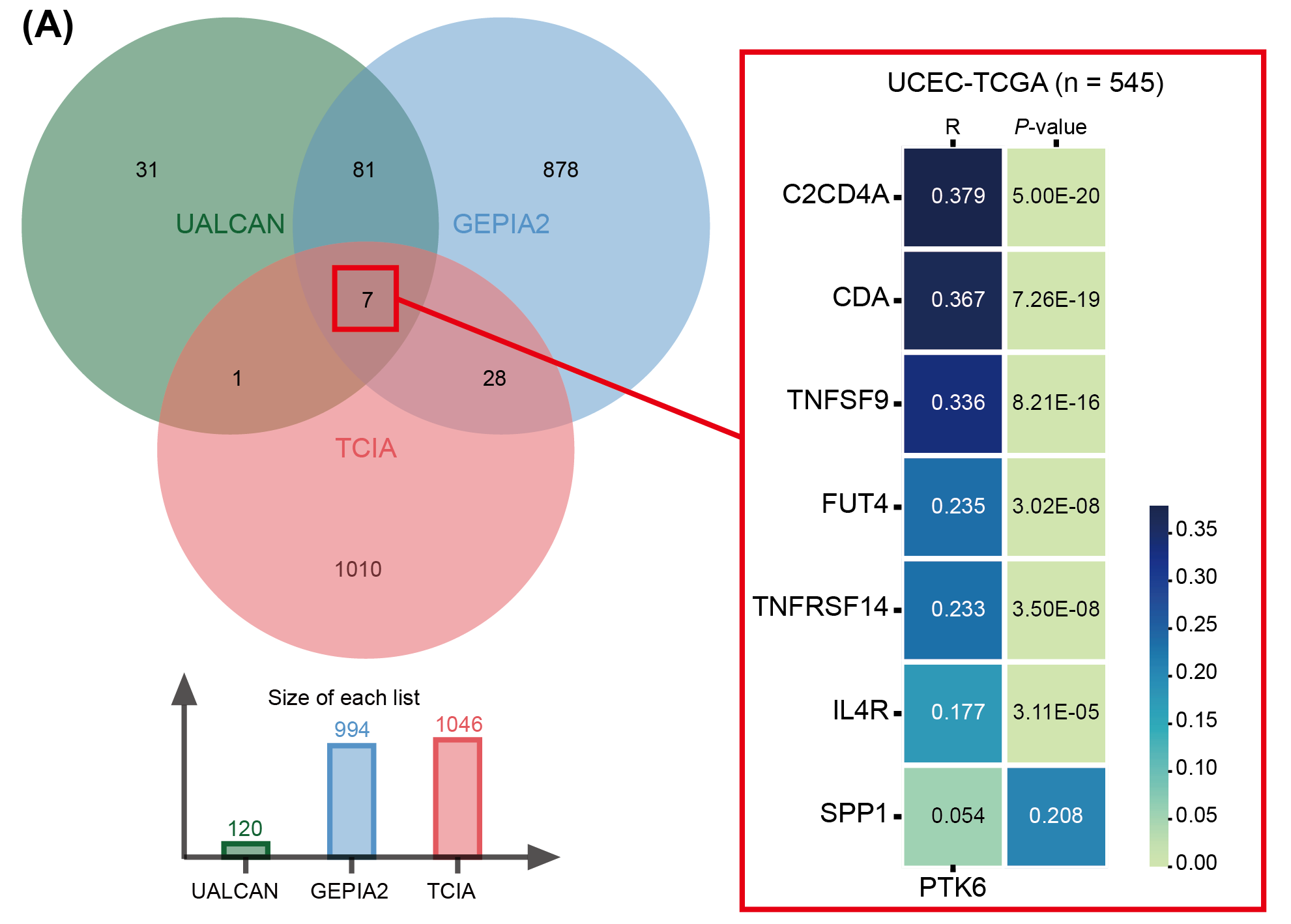

Supplement: Supplementary file 4 [file image_4.tif]

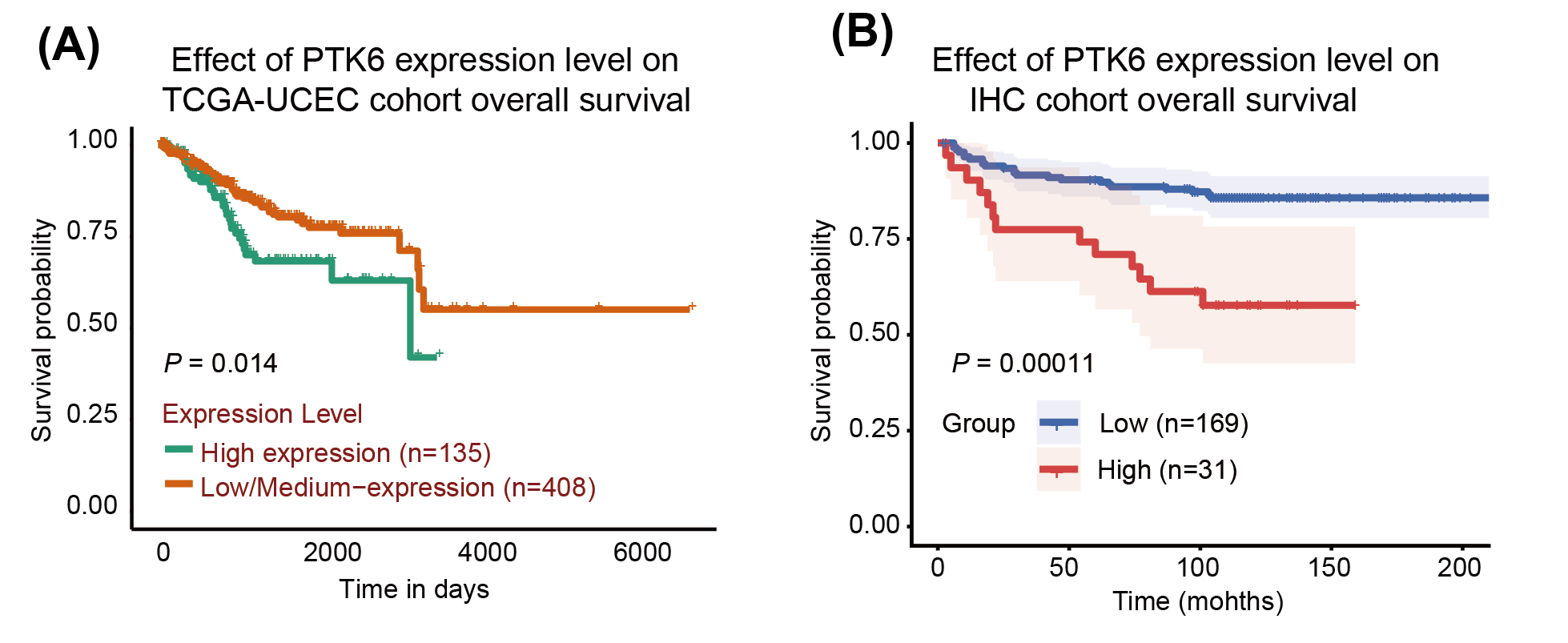

Supplement: Supplementary file 5 [file image_5.tif]
