# Supplementary material for: Elevated PTK6 expression is associated with tumor immune microenvironment remodeling and predicts poor prognosis in endometrial carcinoma
Source: Front Med (Lausanne). 2026 Jun 3;13:1842564. doi: 10.3389/fmed.2026.1842564 (PMC13272333; doi:10.3389/fmed.2026.1842564)
Supplement: Supplementary file 6 [file Supplementary_File_1.docx]

Supplementary Material

# Supplementary Figures

**Supplementary Figure S1.** **Pan-cancer Analysis of PTK6 mRNA Expression in Tumor Tissues Relative to Adjacent Normal Tissues.** PTK6 mRNA was significantly elevated in cancers including BLCA, BRCA, CHOL, KIRC, KIRP, LUAD, LUSC, PRAD, THCA, and UCEC, and significantly reduced in COAD, HNSC and KICH (TCGA data in TIMER, **P* < 0.05, ***P* < 0.01, ****P* < 0.001).

**Supplementary Figure S2.** **Kaplan-Meier Survival Analysis Across All UCEC Molecular Subtypes.** (A) CN-H subtype: OS and PFS. (B) CN-L subtype: OS and PFS. (C) MSI-H subtype: PFS. (D) POLE subtype: OS.

**Supplementary Figure S3. Correlations Between PTK6 Expression and Infiltration Level of immune cells.** Dot size represents the strength of the association between PTK6 and the immune cell type (larger dots corresponding to stronger correlations). Dot color indicates the *P*-value (a more intense red color signifies a smaller *P*-value). *P* < 0.05 was considered significant; red *P*-values indicate significant positive correlations, and blue ones represent significant negative correlations.

**Supplementary Figure S4. Identification of PTK6-Correlated Immune Genes in UCEC.** Venn diagram revealing seven genes consistently coexpressed with PTK6 across three databases: UALCAN, GEPIA2, and TCIA. Significance was confirmed by TIMER3 except for SPP1 (red box; Spearman correlation, *P* < 0.05 was considered significant).

**Supplementary Figure S5. Validation of the prognostic value of PTK6 using independent cutoff strategies.** (A) Kaplan-Meier analysis of PTK6 mRNA for overall survival in the TCGA-UCEC cohort using an independent cutoff from UALCAN. (B) Kaplan-Meier Survival Analysis of PTK6 protein expression using the third-quartile cutoff in the IHC cohort.
